# Inhibition of soluble epoxide hydrolase attenuates renal tubular mitochondrial dysfunction and ER stress by restoring autophagic flux in diabetic nephropathy

**DOI:** 10.1038/s41419-020-2594-x

**Published:** 2020-05-21

**Authors:** Xu-shun Jiang, Xing-yang Xiang, Xue-mei Chen, Jun-ling He, Ting Liu, Hua Gan, Xiao-gang Du

**Affiliations:** 1 Department of Nephrology, The First Affiliated Hospital of Chongqing Medical University, Youyi Road 1, Chongqing, 400042 China; 2 Emergency Department, The First Affiliated Hospital of Chongqing Medical University, Youyi Road 1, Chongqing, 400042 China; 3 Department of Pathology, Leiden University Medical Center, Leiden, The Netherlands; 4 Department of Nephrology, Chengdu Fifth People’s Hospital, Chengdu, 611130 China; 5 The Chongqing Key Laboratory of Translational Medicine in Major Metabolic Diseases, The First Affiliated Hospital of Chongqing Medical University, Youyi Road 1, Chongqing, 400042 China

**Keywords:** Molecular biology, Chronic kidney disease

## Abstract

Diabetic nephropathy (DN) is the leading cause of end-stage renal disease (ESRD), and renal tubular cell dysfunction contributes to the pathogenesis of DN. Soluble epoxide hydrolase (sEH) is an enzyme that can hydrolyze epoxyeicosatrienoic acids (EETs) and other epoxy fatty acids (EpFAs) into the less biologically active metabolites. Inhibition of sEH has multiple beneficial effects on renal function, however, the exact role of sEH in hyperglycemia-induced dysfunction of tubular cells is still not fully elucidated. In the present study, we showed that human proximal tubular epithelial (HK-2) cells revealed an upregulation of sEH expression accompanied by the impairment of autophagic flux, mitochondrial dysfunction, ubiquitinated protein accumulation and enhanced endoplasmic reticulum (ER) stress after high glucose (HG) treatment. Furthermore, dysfunctional mitochondria accumulated in the cytoplasm, which resulted in excessive reactive oxygen species (ROS) generation, Bax translocation, cytochrome c release, and apoptosis. However, *t*-AUCB, an inhibitor of sEH, partially reversed these negative outcomes. Moreover, we also observed increased sEH expression, impaired autophagy flux, mitochondrial dysfunction and enhanced ER stress in the renal proximal tubular cells of db/db diabetic mice. Notably, inhibition of sEH by treatment with *t*-AUCB attenuated renal injury and partially restored autophagic flux, improved mitochondrial function, and reduced ROS generation and ER stress in the kidneys of db/db mice. Taken together, these results suggest that inhibition of sEH by *t*-AUCB plays a protective role in hyperglycemia-induced proximal tubular injury and that the potential mechanism of *t*-AUCB-mediated protective autophagy is involved in modulating mitochondrial function and ER stress. Thus, we provide new evidence linking sEH to the autophagic response during proximal tubular injury in the pathogenesis of DN and suggest that inhibition of sEH can be considered a potential therapeutic strategy for the amelioration of DN.

## Introduction

Diabetic nephropathy (DN) is a common and serious microvascular complication of diabetes mellitus (DM), and it is the leading cause of end-stage renal disease (ESRD)^1,2^. Numerous lines of evidence have demonstrated that renal tubular cell injury plays a critical role in the pathogenesis and progression of DN and it has been recognized as a reliable predictor of renal functional deterioration and a hallmark of DN^3,4^. Therefore, protecting renal tubular cells from injury is an effective strategy for slowing down the development of DN.

Autophagy is an evolutionarily conserved catabolic process in which various intracellular components, such as unfolded/misfolded proteins and damaged organelles, are delivered to lysosomes for degradation, clearance and recycling^5^. A basal level of autophagy is required for cells to maintain intracellular homeostasis, whereas stress-induced autophagy primarily serves as an adaptive and defensive mechanism for cell survival. Emerging evidence suggests that autophagy is involved in the pathogenesis of diverse diseases, including cardiovascular diseases^6^, aging and neurodegenerative disease^7^, cancers^8^, and infectious and inflammatory disease^9^. A growing number of studies have indicated that autophagy contributes to the pathogenesis of many important kidney diseases such as acute kidney injury (AKI)^10^, lupus nephritis^11^, polycystic kidney disease (PKD)^12^, and DN^13^. Additionally, autophagy is crucial for maintaining renal homeostasis and health, and insufficient autophagy is likely to be involved in the vulnerability of renal tubular cells, leading to severe tubular cell damage and the rapid progression of DN^14^. Meanwhile, other studies have shown that impaired autophagy may lead to mitochondrial dysfunction and increased ER stress in DN^15,16^. Therefore, restoring autophagy activity may be a potential therapeutic strategy for DN. However, the exact role that autophagy plays in the renal tubular cells of DN is still not fully elucidated.

Epoxyeicosatrienoic acids (EETs), which are metabolized from arachidonic acid by cytochrome P450 (CYP) enzymes, play a crucial role in the regulation of inflammation, vascular remodeling, hypertension, and organ and tissue regeneration^17^. However, EETs are rapidly hydrolyzed by soluble epoxide hydrolase (sEH) into the less biologically active metabolite, dihydroxyeicosatrienoic acid (DHET)^18^. sEH is a cytosolic enzyme that is widely distributed in the liver, heart and kidney, and it plays a pivotal role in the regulation of EET bioavailability^19^. Numerous studies have highlighted the potential benefits of sEH inhibition in the inflammatory response^20^, cardiovascular diseases^21^, non-alcoholic fatty liver^22^ and renal disease^23–25^. A recent study demonstrated that the sEH inhibitor TUPS mitigated isoproterenol/angiotensin II-induced cardiac hypertrophy by inhibiting mTOR signaling-mediated autophagy^26^, which indicated that sEH is associated with the regulation of autophagy. Furthermore, previous evidence demonstrated that the stabilization of EpFA by treatment with an sEH inhibitor prevented mitochondrial dysfunction, which subsequently reduced ROS generation and blocked the activation of ER stress in diverse diseases including diabetes, cardiovascular and neurodegenerative diseases^27^. Nonetheless, the underlying mechanisms of sEH inhibition in hyperglycemia-induced renal injury and the relationship among mitochondrial dysfunction, ER stress and autophagy remain poorly understood.

Given the potential role of sEH inhibition in DN, the aim of this study was to evaluate the effects of the sEH inhibitor *t*-AUCB on proximal tubular injury in the kidneys of db/db mice and clarify the possible mechanisms underlying the regulation of mitochondrial dysfunction, ER stress and autophagy flux by *t*-AUCB in HK-2 cells exposed to HG conditions.

## Results

### HG conditions increased sEH expression and induced impaired autophagy in HK-2 cells

**Fig. 1:**
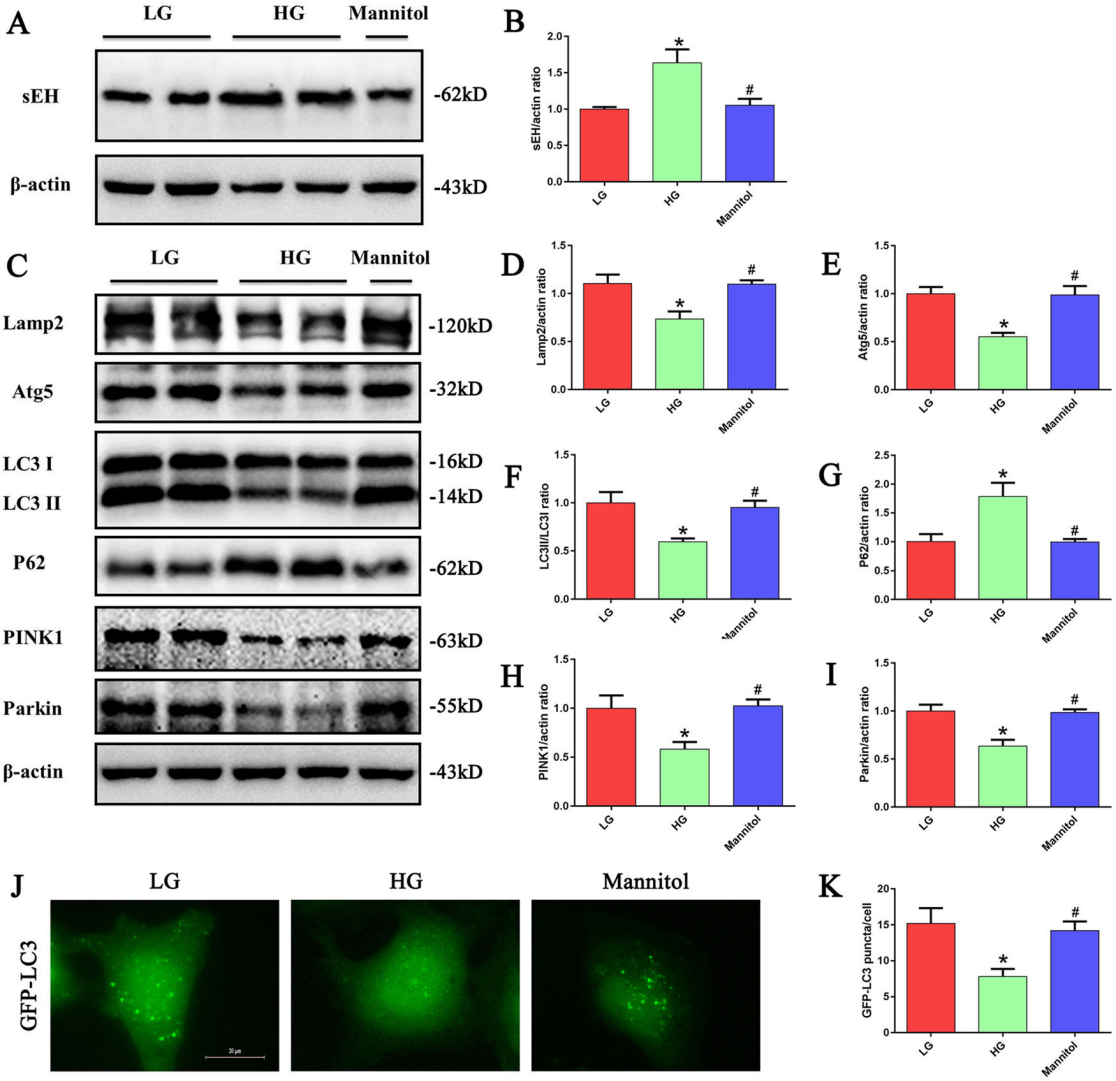
HG treatment increased sEH expression and induced impaired autophagy in HK-2 cells. **a** Western blot analysis of sEH expression in HK-2 cells treated with LG, HG or Mannitol for 24 h. **b** Densitometric analysis of sEH. (*n* = 3, *P < 0.05 vs. LG, ^#^*P* < 0.05 vs. HG.) **c** Western blot analysis of Lamp2, Atg5,LC3-II/LC3-I, p62, PINK1and Parkin expression in HK-2 cells treated with LG, HG or Mannitol for 24 h. **d–i** Densitometric analysis of Lamp2, Atg5,LC3-II/LC3-I, p62, PINK1 and Parkin (*n* = 3, *P < 0.05 vs. LG, ^#^*P* < 0.05 vs. HG). **j** HK-2 cells transfected with GFP-LC3 were treated with LG, HG or Mannitol for 24 h, and then observed for the change of green fluorescence using a fluorescence microscope. **k** Quantification of the number of GFP-LC3 dots in HK-2 cells (*n* = 5, *P < 0.05 vs. LG, ^#^*P* < 0.05 vs. HG).

We first investigated the expression of sEH in HK-2 cells under HG stimulation, HK-2 cells were treated with low- glucose (LG, 5 mM), high- glucose (HG, 30 mM), or mannitol (30 mM) for 24 h. We found that compared to the other conditions, HG exposure significantly increased sEH protein expression in HK-2 cells (Fig. 1a). The role of HG in the autophagy of HK-2 cells was further investigated in the current study. Immunoblot analyses revealed decreased expression of lysosomal-associated membrane protein 2 (Lamp2), autophagy-related gene 5 (Atg5), and LC3-II/LC3-I, whereas p62 protein, a selective substrate of autophagy, was upregulated (Fig. 1c). In addition, the expression of the mitophagy-related proteins PTEN-induced putative kinase 1 (PINK1) and Parkin was decreased (Fig. 1c). Moreover, as LC3 is a major constituent of the autophagosome, we established a stable GFP-LC3-expressing HK-2 cell line to visualize autophagosome formation in HK-2 cells. We found that the number of GFP-LC3 puncta was reduced in HG treated cells compared to the control (Fig. 1j).

### HG conditions induced mitochondrial damage, ER stress, and apoptosis in HK-2 cells

**Fig. 2:**
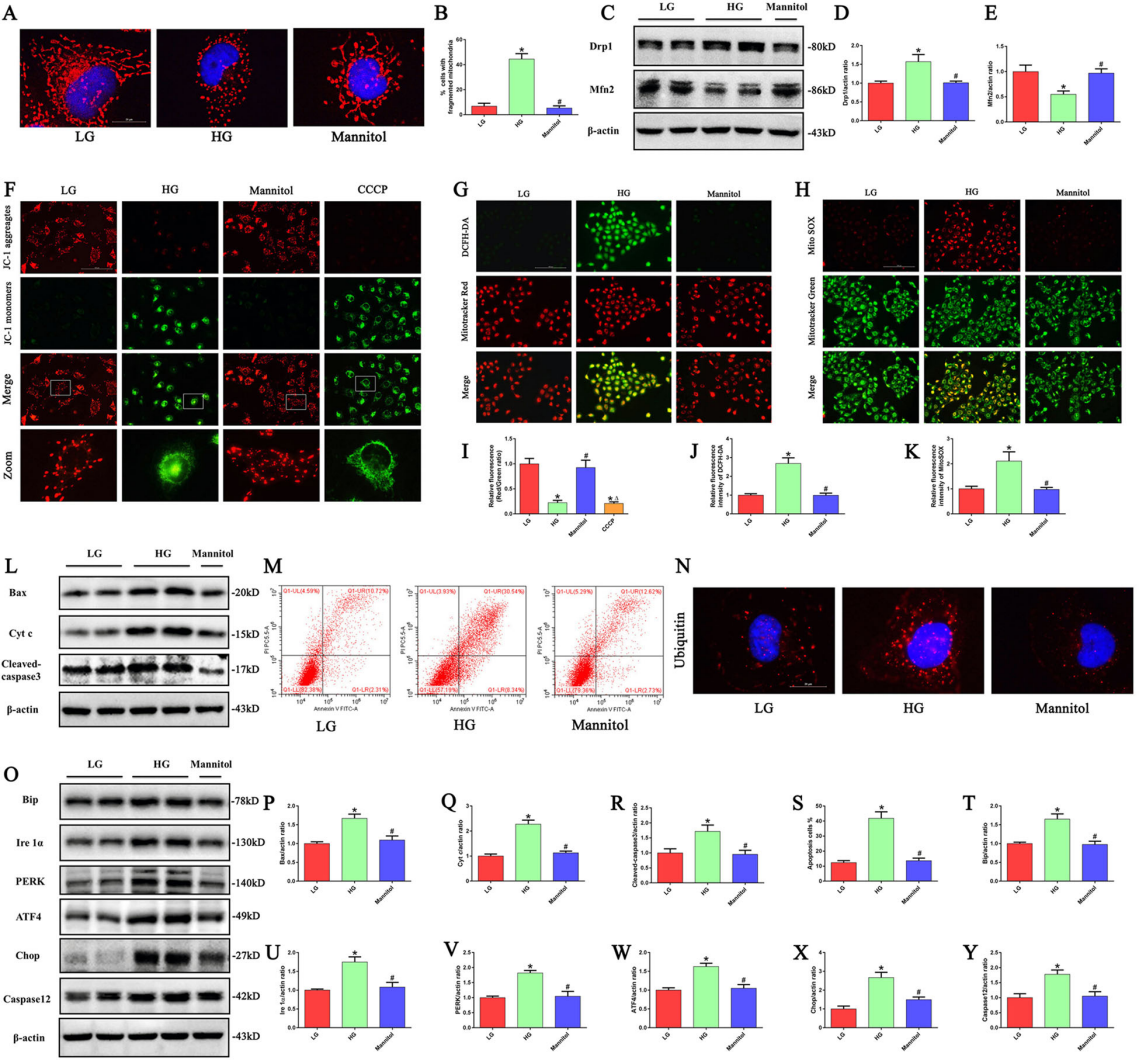
HG induced mitochondrial damage, ROS generation, ER stress, and apoptosis in HK-2 cells. **a** Representative images of MitoTracker Red staining showing the mitochondrial morphology in HK-2 cells treated with LG, HG or Mannitol for 24 h. **b** Quantification of percentage of HK-2 cells with fragmented mitochondria (*n* = 4, *P < 0.05 vs. LG, ^#^*P* < 0.05 vs. HG). **c** Western blot analysis of Drp1, Mfn2 in HK-2 cells treated with LG, HG or Mannitol for 24 h. **d**, **e** Densitometric analysis of Drp1and Mfn2 (*n* = 3, *P < 0.05 vs. LG, ^#^*P* < 0.05 vs. HG). **f** Representative images of JC-1 staining showing the mitochondrial membrane potential in HK-2 cells treated with LG, HG, Mannitol or CCCP. **g** HK-2 cells were treated with LG, HG or Mannitol for 24 h, and then incubated with DCFH-DA and MitoTracker Red. **h** HK-2 cells were treated with LG, HG or Mannitol for 24 h, and then incubated with Mito SOX and MitoTracker Green. **i–k** Quantification of the fluorescence intensity of JC-1, DCFH-DA and Mito SOX staining in figures f–h (*n* = 3, *P < 0.05 vs. LG, ^#^*P* < 0.05 vs. HG). **l** Western blot analysis of Bax, Cyt c and cleaved-caspase3 in HK-2 cells treated with LG, HG or Mannitol for 24 h. **m** Flow cytometry analysis of the cell apoptotic rate. (**n**) Representative images of immunofluorescence staining of ubiquitin in HK-2 cells. **o** Western blot analysis of Bip, Ire1α, PERK, ATF4, Chop, Caspase12 in HK-2 cells treated with LG, HG or Mannitol for 24 h. **p–r** Densitometric analysis of Bax, Cyt c and cleaved-caspase3 in figure l (*n* = 3, *P < 0.05 vs. LG, ^#^*P* < 0.05 vs. HG). **s** Quantification of the cell apoptotic rate in figure m (*n* = 3, *P < 0.05 vs. LG, ^#^*P* < 0.05 vs. HG). **t–y** Densitometric analysis of Bip, Ire1α, PERK, ATF4, Chop, Caspase12 in figure o (*n* = 3, *P < 0.05 vs. LG, ^#^*P* < 0.05 vs. HG).

The effect of HG treatment on mitochondrial morphological changes in HK-2 cells was further confirmed by immunofluorescence staining with MitoTracker Red. In the control cells, mitochondria exhibited a filamentous shape, whereas they transformed into short rod shapes after HG treatment for 24 h (Fig. 2a). In addition, HG treatment significantly increased the expression of the mitochondrial fission protein Drp1 but decreased the expression of the mitochondrial fusion protein Mfn2 (Fig. 2c). JC-1 staining showed that HG induced a reduction in mitochondrial membrane potential (ΔΨm) (Fig. 2f), and it was accompanied by increased intracellular and mitochondrial ROS production after HG treatment (Fig. 2g, h). Meanwhile, HG treatment induced an increase in Bax, cytochrome c (Cyt c) and cleaved-caspase3 protein expression and promoted apoptosis of HK-2 cells (Fig. 2l, m).

The accumulation of misfolded proteins in the ER can lead to ER stress, which subsequently triggers a specialized response known as the unfolded protein response (UPR)^16^. As shown in Fig. 2n, we found that HG induced significant ubiquitinated protein accumulation in HK-2 cells. In addition, HG induced enhanced ER stress in HK-2 cells, as evidenced by increased expression of ER stress- related proteins, such as Bip, inositol-requiring enzyme 1 (Ire1α), PKR-like ER-regulated kinase (PERK), activating transcription factor 4 (ATF4), Chop and Caspase12 (Fig. 2o).

### Inhibition of sEH restored HG induced impaired autophagy flux and mitophagy

**Fig. 3:**
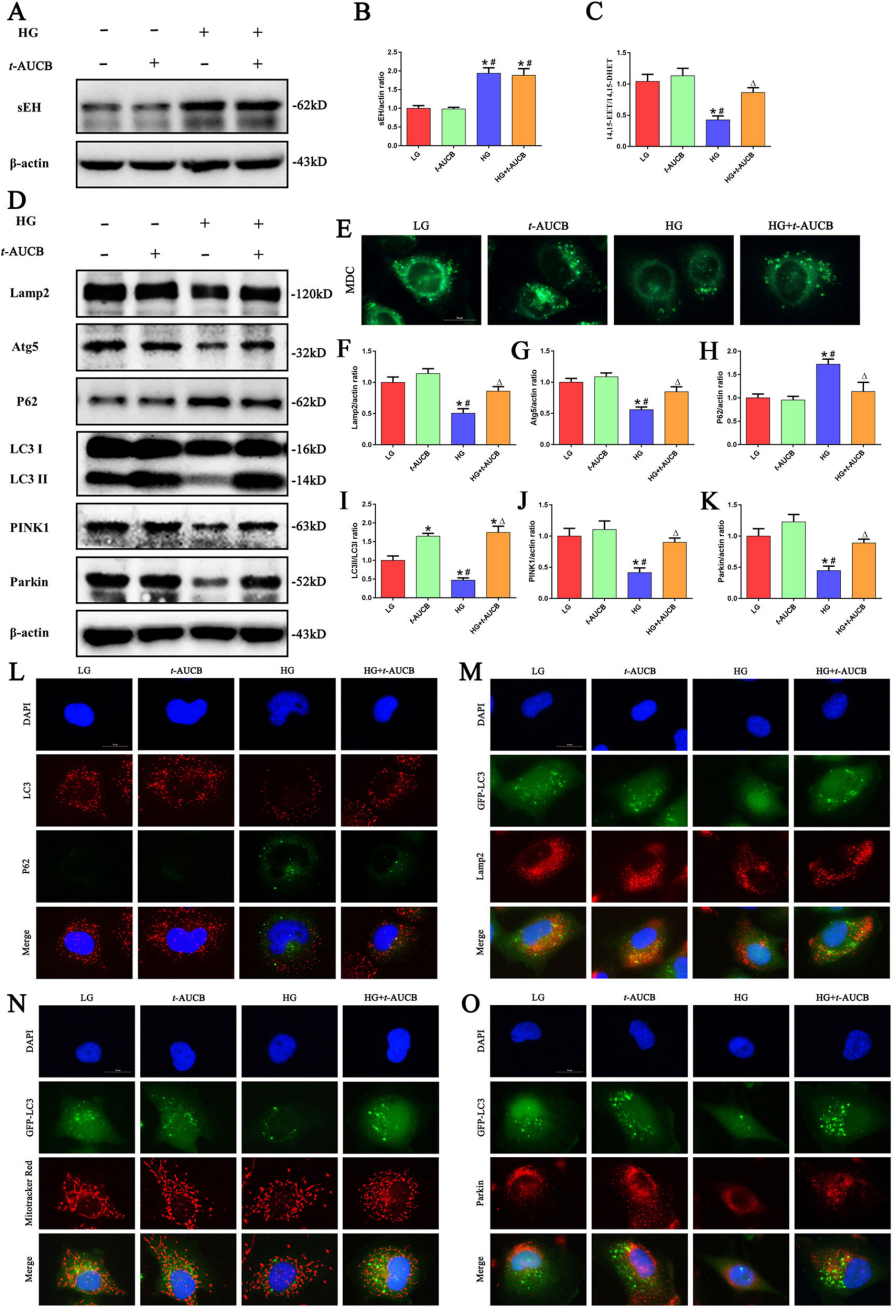
Inhibition of sEH restored HG -induced impaired autophagy flux and mitophagy. **a** Western blot analysis of sEH in HK-2 cells treated with HG or/and *t*-AUCB for 24 h. **b** Densitometric analysis of sEH (*n* = 3, *P < 0.05 vs. LG, ^#^*P* < 0.05 vs. *t*-AUCB). **c** ELISA kit analysis of sEH enzymatic activity (14,15-EET to 14,15-DHET ratios) in HK-2 cells (*n* = 3, *P < 0.05 vs. LG, ^#^*P* < 0.05 vs. *t*-AUCB, ^Δ^*P* < 0.05 vs. HG). **d** Western blot analysis of Lamp2, Atg5, LC3-II/LC3-I, p62, PINK1 and Parkin in HK-2 cells treated with HG or/and *t*-AUCB for 24 h. **e** Representative images of MDC staining in different groups of HK-2 cells after treated with HG or/and *t*-AUCB for 24 h. **f–k** Densitometric analysis of Lamp2, Atg5,LC3-II/LC3-I, p62, PINK1 and Parkin in figure d (*n* = 3, *P < 0.05 vs. LG, ^#^*P* < 0.05 vs. *t*-AUCB, ^Δ^*P* < 0.05 vs. HG). **l** Representative images of immunofluorescence double labeling of LC3 and P62 in different groups of HK-2 cells after treated with HG or/and *t*-AUCB for 24 h. **m–o** Colocalization analysis of immunofluorescence images of GFP-LC3 (green) and Lamp2 (red), GFP-LC3 (green) and Mito Tracker (red), GFP-LC3 (green) and Parkin (red) in different groups of HK-2 cells after treated with HG or/and *t*-AUCB for 24 h.

To determine the effect of *t*-AUCB, an sEH inhibitor, on sEH protein expression in HK-2 cells exposed to HG, Western blot analyses were performed. We found that *t*-AUCB did not significantly affect the protein expression of sEH (Fig. 3a). The sEH mainly functions as a hydrolase enzyme; therefore, we measured the effect of *t*-AUCB on its enzymatic activity by determining the 14,15-EET/14,15-DHET ratio in HK-2 cells. As expected, *t*-AUCB significantly inhibited sEH enzymatic activity, which was revealed by an increase in the 14,15-EET/14,15-DHET ratio value in HK-2 cells under HG treatment (Fig. 3c). Meanwhile, we found that *t*-AUCB significantly increased the levels of 14,15-EET and decreased the levels of 14,15-DHET in HK-2 cells under HG treatment (Supplement Fig. 1a, b).

To further investigate the effect of *t*-AUCB on autophagy in HK-2 cells, autophagy-related proteins were assessed by Western blot. As shown in Fig. 3d, HG stimulation significantly decreased the expression of Lamp2, Atg5, the LC3 II/I ratio, PINK1 and Parkin, and it increased the p62 protein expression in HK-2 cells, while these changes were significantly prevented following *t*-AUCB treatment. Moreover, monodansylcadaverine (MDC) staining showed that *t*-AUCB treatment increased the number of autophagic vacuoles in HG-induced HK-2 cells (Fig. 3e). Next, to precisely monitor autophagic flux, double immunofluorescence for LC3 and p62 was performed. Under HG conditions, HK-2 cells exhibited a reduced number of punctate LC3 dots and enhanced p62 fluorescence intensity; However, cells treated with *t*-AUCB improved autophagy flux, which was evidenced by the increased number of punctate LC3 dots and decreased p62 fluorescence intensity (Fig. 3l). Moreover, we observed that *t*-AUCB treatment increased the colocalization of GFP-LC3 with Lamp2 in HK-2 cells under HG stimulation, suggesting that *t*-AUCB increased the fusion of autophagosomes with lysosomes in HK-2 cells (Fig. 3m). Furthermore, immunofluorescence for GFP-LC3 and MitoTracker Red, GFP-LC3 and Parkin staining was used to analyze mitophagy, and the results showed that *t*-AUCB treatment increased the colocalization of GFP-LC3 with mitochondria or Parkin in HK-2 cells under HG stimulation, indicating that *t*-AUCB improved HG -induced mitophagy (Fig. 3n, o).

### Inhibition of sEH attenuated HG induced mitochondrial dysfunction, ER stress and apoptosis in HK-2 cells

**Fig. 4:**
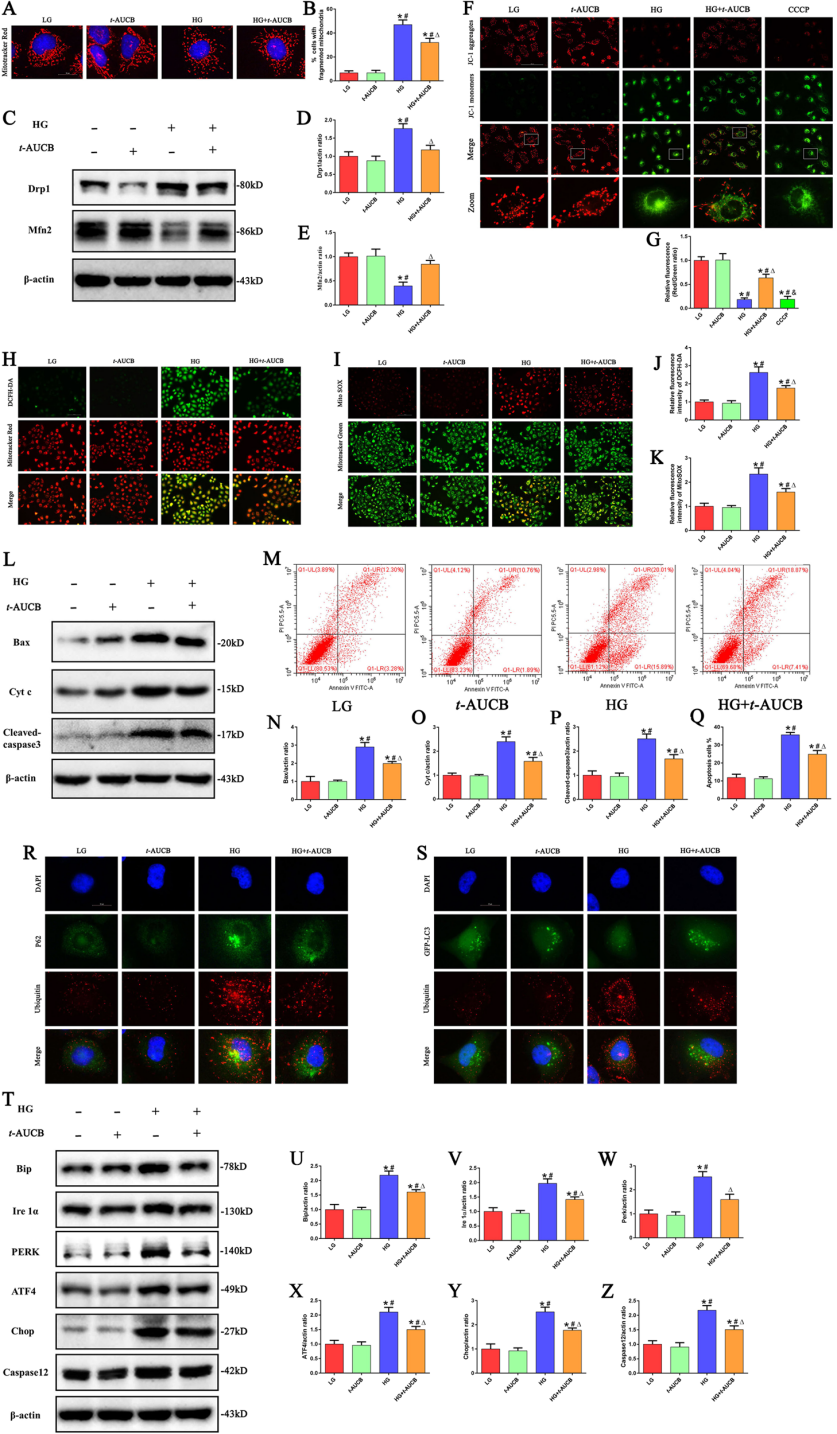
Inhibition of sEH attenuated HG induced mitochondrial dysfunction, ER stress and apoptosis in HK-2 cells. **a** Representative images of MitoTracker Red staining showing the mitochondrial morphology in HK-2 cells after treated with HG or/and *t*-AUCB for 24 h. **b** Quantification of percentage of HK-2 cells with fragmented mitochondria (*n* = 4, *P < 0.05 vs. LG, ^#^*P* < 0.05 vs. *t*-AUCB, ^Δ^*P* < 0.05 vs. HG). **c** Western blot analysis of Drp1, Mfn2 in HK-2 cells after treated with HG or/and *t*-AUCB for 24 h. **d**, **e** Densitome*t*ric analysis of Drp1 and Mfn2 (*n* = 3, *P < 0.05 vs. LG, ^#^*P* < 0.05 vs. *t*-AUCB, ^Δ^*P* < 0.05 vs. HG). **f** Representative images of JC-1 staining showing the ΔΨm in different groups of HK-2 cells. **g** Quantification of the fluorescence intensity of JC-1 staining (*n* = 3, *P < 0.05 vs. LG, ^#^*P* < 0.05 vs. *t*-AUCB, ^Δ^*P* < 0.05 vs. HG, ^&^*P* < 0.05 vs. HG + *t*-AUCB). **h, i** HK-2 cells were treated with HG or/and *t*-AUCB for 24 h, and then incubated with DCFH-DA and Mi*t*oTracker Red, or Mito SOX and MitoTracker Green. **j**, **k** Quantification of the fluorescence intensity of DCFH-DA and Mito SOX staining in figures h and i (*n* = 3, *P < 0.05 vs. LG, ^#^*P* < 0.05 vs. *t*-AUCB, ^Δ^*P* < 0.05 vs. HG). **l** Western blot analysis of Bax, Cyt c and cleaved-caspase3 in HK-2 cells after treated with HG or/and *t*-AUCB for 24 h. **m** Flow cytometry analysis of the cell apoptotic rate. **n–p** Densitometric analysis of Bax, Cyt c and cleaved-caspase3 in figure l. **q** Quantification of the cell apoptotic rate in figure m (*n* = 3, *P < 0.05 vs. LG, ^#^*P* < 0.05 vs. t-AUCB, ^Δ^*P* < 0.05 vs. HG). **r** Representative images of immunofluorescence double-labeling P62 and Ubiquitin in different groups of HK-2 cells after treated with HG or/and *t*^-^AUCB for 24 h. **s** Colocalization analysis of immunofluorescence images of GFP-LC3 (green) and Ubiquitin (red) in different groups of HK-2 cells after treated with HG or/and *t*-AUCB for 24 h. **t** Western blot analysis of Bip, Ire1α, PERK, ATF4, Chop, Caspase12 in HK-2 cells after treated with HG or/and *t*-AUCB for 24 h. **u–z** Densitometric analysis of Bip, Ire1α, PERK, ATF4, Chop and Caspase12 in figure t (*n* = 3, *P < 0.05 vs. LG, ^#^*P* < 0.05 vs. *t*-AUCB, ^Δ^*P* < 0.05 vs. HG).

Next, we wanted to provide further evidence that the inhibition of sEH with *t*-AUCB affects mitochondrial function in HG-induced HK-2 cells. First, we found that *t*-AUCB significantly attenuated the percentages of fragmented mitochondria under HG stimulation (Fig. 4a), which was accompanied by increased expression of Mfn2 and reduced the levels of Drp1 (Fig. 4c). In addition, *t*-AUCB treatment significantly reversed the loss of the ΔΨm (Fig. 4f) and further attenuated the production of intracellular and mitochondrial ROS (Fig. 4h, i) in HK-2 cells under HG conditions. Moreover, *t*-AUCB markedly decreased the expression of Bax, Cyt c and cleaved- caspase3 protein expression in HG-induced HK-2 cells (Fig. 4l). Furthermore, immunofluorescence showed that HG treatment induced Bax translocation from the cytosol to the mitochondria (Supplement Fig. 2a) and enhanced the release of Cyt c from the mitochondria to the cytosol (Supplement Fig. 2b), suggesting that HG caused Bax and Cyt c redistribution. However, these changes were partially attenuated in HK-2 cells treated with *t*-AUCB, subsequently leading to attenuated cell apoptosis (Fig. 4m).

It has been reported that ubiquitinated proteins can be degraded by autophagy mediated via receptor proteins (e.g., p62/SQSTM-1); thus autophagy can be an adaptive cellular mechanism to combat ER stress and eliminate misfolded proteins^28^. As shown in Fig. 4r, we found that the colocalization of p62 with ubiquitinated aggregates existed in HK-2 cells under HG treatment. However, HG-induced impaired autophagy flux hinders autophagic function as a compensatory mechanism to remove misfolded protein aggregates. Interestingly, we found that *t*-AUCB treatment significantly increased the colocalization of GFP-LC3 puncta with ubiquitinated aggregates and attenuated the accumulation of ubiquitinated proteins in HK-2 cells under HG stimulation (Fig. 4s). Furthermore, we also found that *t*-AUCB significantly attenuated the expression of ER stress-related proteins, such as Bip, Ire1α, PERK, ATF4, Chop and Caspase12 (Fig. 4t), indicating that *t*-AUCB attenuated HG-induced ER stress via the autophagy pathway in HK-2 cells.

### Effect of the sEH inhibitor *t*-AUCB on renal functional and morphologic characteristics in db/db mice

**Fig. 5:**
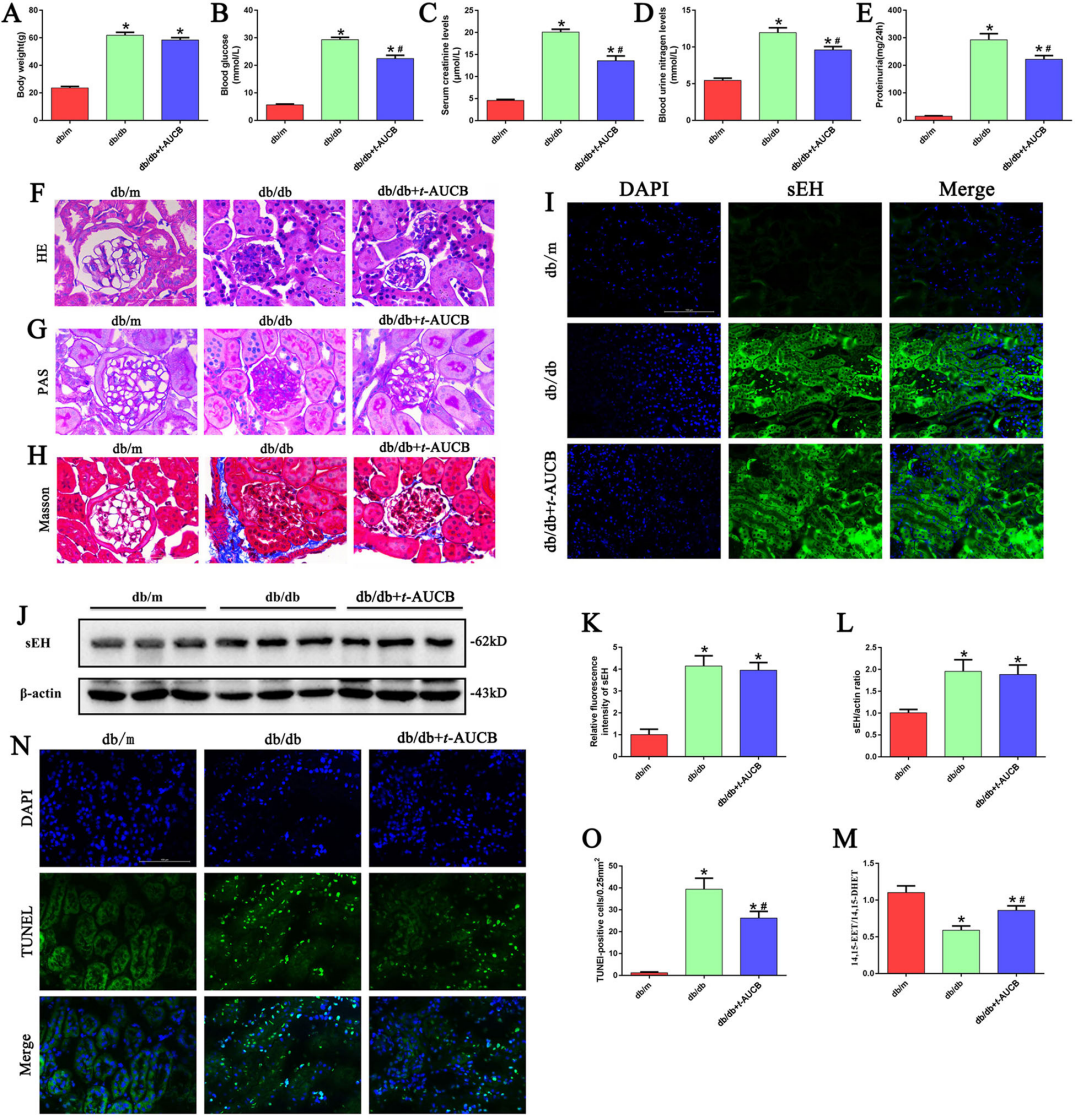
Effect of sEH inhibitor *t*-AUCB on renal functional and morphologic characteristics in db/db mice. The changes of body weight (**a**), blood glucose levels (**b**), serum creatinine (SCr) levels (**c**), blood urea nitrogen (BUN) levels (**d**), urine protein levels (**e**) in db/m, db/db and db/db mice treated with *t*-AUCB. **f–h** Kidney sections stained with H&E, PAS, MassonTrichrome. **i** Representative images of immunofluorescence staining of sEH in kidney tissues. **j** Western blot analysis of sEH expression in kidney tissues. **k** Quantification of the fluorescence intensity of sEH in figure i. **l** Densitometric analysis of sEH expression in figure j. **m** ELISA kit analysis of 14,15-EET to 14,15-DHET ratios in the urine of db/m, db/db and db/db mice treated with *t*-AUCB. **n–o** Representative images and quantification of TUNEL staining of kidney tissues. *P < 0.05 vs. db/m, ^#^*P* < 0.05 vs. db/db.

Next, we examined whether inhibition of sEH with *t*-AUCB ameliorated renal damage in db/db mice. No significant difference was found in body weight between the db/db mice treated with *t*-AUCB and the untreated db/db mice (Fig. 5a). Compared with control mice, the db/db mice had higher blood glucose levels (Fig. 5b) and increased serum creatinine (Fig. 5c), blood urea nitrogen (Fig. 5d) and urine protein levels (Fig. 5e). However, all of these increased parameters were significantly attenuated following *t*-AUCB treatment. Further analysis demonstrated that glomerular mesangial matrix accumulation and renal fibrosis were enhanced in db/db mice compared with that of the control mice (Fig. 5f–h). However, these pathological changes in db/db mice were partially improved with *t*-AUCB treatment. In addition, immunofluorescence and Western blot analysis showed that the level of sEH expression in the kidneys of db/db mice was significantly higher than that in control mice. However, treatment with *t*-AUCB did not affect the expression of sEH in the kidneys of db/db mice compared with untreated db/db mice (Fig. 5i, j). Moreover, *t*-AUCB dramatically inhibited sEH enzymatic activity, which was manifested as an increase in the 14,15-EET/14,15-DHET ratio (Fig. 5m). Meanwhile, *t*-AUCB treatment significantly increased the levels of 14,15-EET and decreased the levels of 14,15-DHET in the urine of db/db mice compared with untreated db/db mice (Supplement Fig. 3a, b). Furthermore, TUNEL staining demonstrated that the number of positive cells was markedly increased in the kidneys of db/db mice, and this increase was attenuated by *t*-AUCB treatment (Fig. 5n). Taken together, our data demonstrated that *t*-AUCB treatment had a beneficial effect on the renal function of db/db mice.

### Inhibition of sEH with *t*-AUCB restored autophagy flux and mitophagy in the proximal tubules of db/db mice

**Fig. 6:**
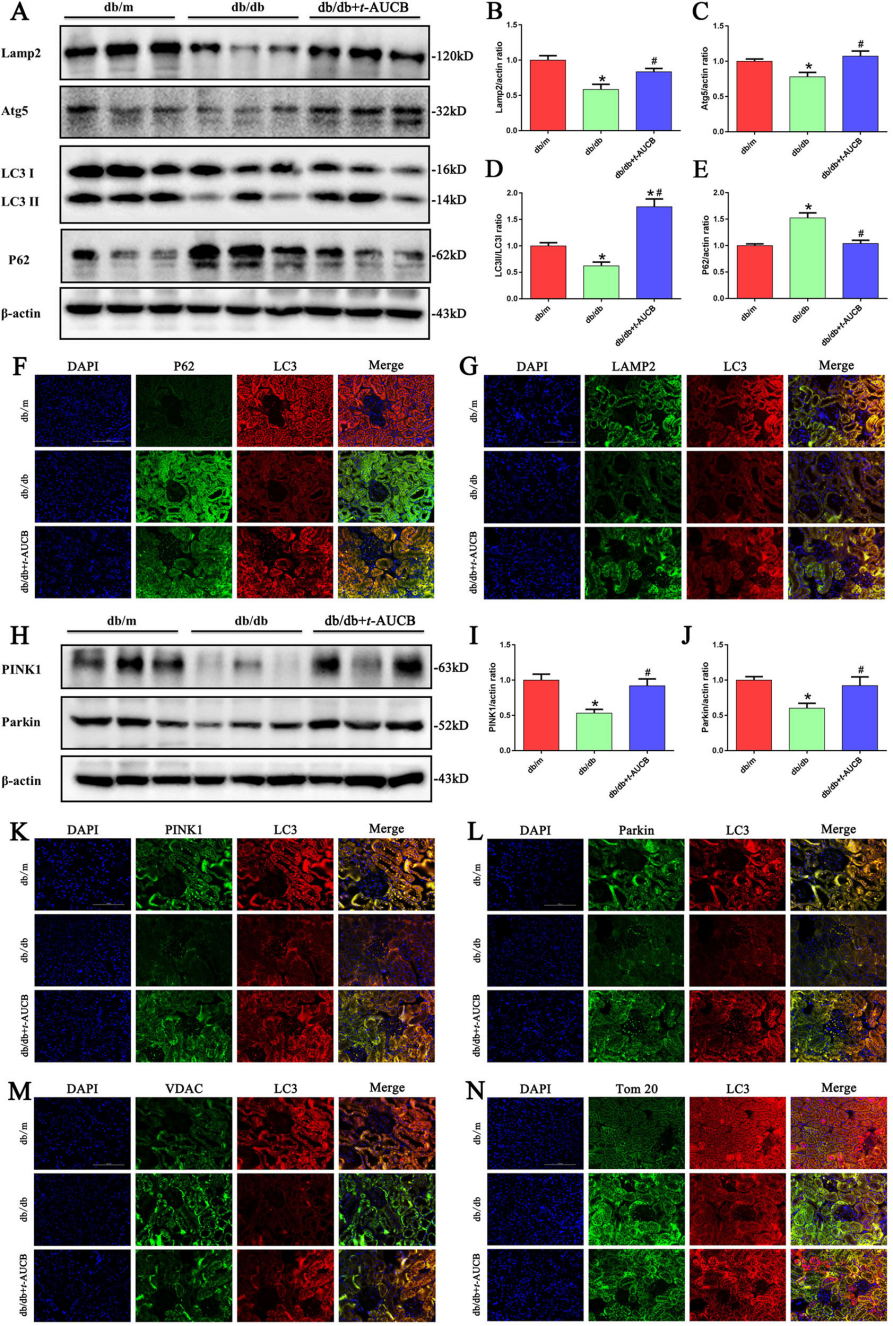
Inhibition of sEH with *t*-AUCB restored autophagy flux and mitophagy in tubules of db/db mice. **a** Western blot analysis of Lamp2, Atg5, LC3-II/LC3-I and p62 expression in kidney tissues. **b–e** Densitometric analysis of Lamp2, Atg5, LC3-II/LC3-I and p62 expression. **f–g** Representative images of immunofluorescence double labeling of P62 and LC3, or Lamp2 and LC3 in different kidney tissues. **h** Western blot analysis of PINK1and Parkin expression in kidney tissues. **i, j** Densitometric analysis of PINK1 and Parkin expression. **k–n** Representative images of immunofluorescence double labeling of PINK1 and LC3, Parkin and LC3, VDAC and LC3,Tom20 and LC3 in different kidney tissues. *P < 0.05 vs. db/m, ^#^*P* < 0.05 vs. db/db.

Autophagy is an adaptive response, and it plays a critical role in the pathogenesis of DN. In db/db mice, we found a significant decrease in the expression of Lamp2, Atg5, and the LC3 II/I ratio, and an elevation of the p62 protein expression. Interestingly, *t*-AUCB administration increased the expression of lamp2 and Atg5, and the LC3 II/I ratio and reduced p62 expression levels (Fig. 6a). Similarly, the alteration of autophagic flux in the proximal tubules of the db/db mice was further confirmed using double fluorescent immunostaining for LC3 and p62, LC3 and Lamp2 (Fig. 6f, g).

To accurately monitor mitophagy, the related regulatory proteins PINK1 and Parkin were detected by Western blot and immunofluorescence analysis, and we found that the kidneys from db/db mice exhibited decreased expression levels of PINK1 and Parkin; however, *t*-AUCB treatment upregulated the expression levels of PINK1 and Parkin (Fig. 6h) and increased the colocalization of PINK1 and LC3 (Fig. 6k) as well as Parkin and LC3 (Fig. 6l). Furthermore, using fluorescent immunostaining, we examined the biochemical hallmarks of mitophagy by measuring the levels of the following mitochondrial proteins: the mitochondrial outer membrane voltage-dependent anion channel (VDAC) and translocase of outermitochondrial membrane 20 (Tom20). We found that the levels of VDAC and Tom20 were significantly increased in the tubules of db/db mice (Fig. 6m, n), indicating that mitophagy deficiency results in the blocked degradation of mitochondrial outer membrane proteins and impairment of mitochondrial recycling. However, *t*-AUCB treatment decreased the expression levels of VDAC and Tom20. Meanwhile, the double immunofluorescence staining showed that *t*-AUCB administration increased the colocalization of LC3 and VDAC or Tom20 (Fig. 6m, n), suggesting upregulation of mitophagy. Collectively, these results suggested that autophagic flux is impaired in db/db mice, and inhibition of sEH with *t*-AUCB restored autophagic impairment and enhanced the process of mitophagy through the upregulation of PINK1 and Parkin.

### *t*-AUCB administration attenuated mitochondrial dysfunction, ROS production, and apoptosis in the proximal tubules of db/db mice

**Fig. 7:**
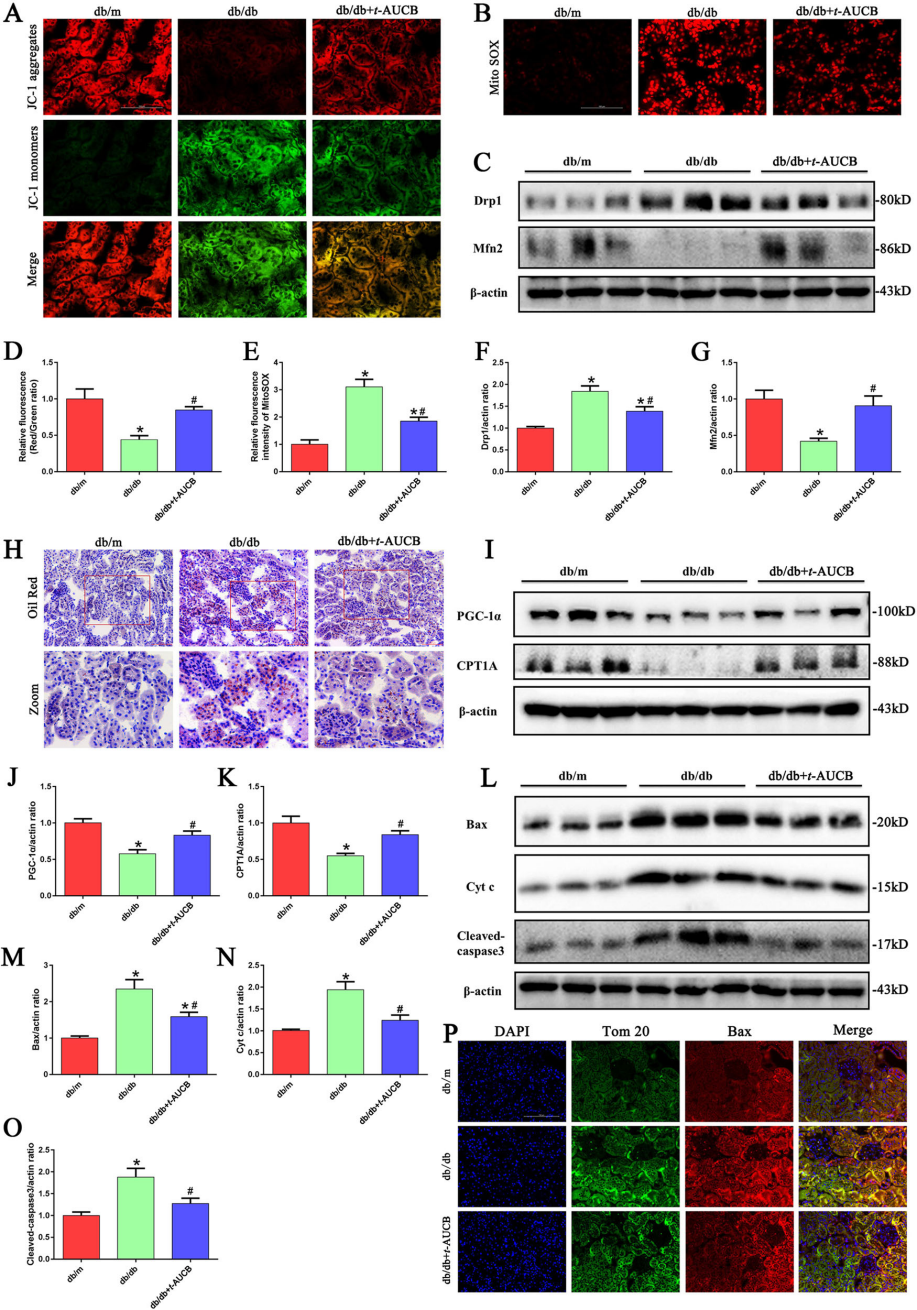
*t*-AUCB administration attenuated mitochondrial dysfunction, ROS production, and apoptosis in tubules of db/db mice. **a** Kidney sections stained with JC-1. **b** Kidney sections stained with Mito SOX. **c** Western blot analysis of Drp1and Mfn2 expression in kidney tissues. **d** Quantification of the fluorescence intensity of JC-1 staining in figure a. **e** Quantification of the fluorescence intensity of Mito SOX staining in figure b. **f**, **g** Densitometric analysis of Drp1and Mfn2 expression in the figure c. **h** Kidney sections stained with Oil Red O staining. **i** Western blot analysis of PGC-1α and CPT1A expression in kidney tissues. **j**, **k** Densitometric analysis of PGC-1α and CPT1A expression in figure i. **l** Western blot analysis of Bax, Cyt c and cleaved-caspase3 expression in kidney tissues. **m–o** Densitometric analysis of Bax, Cyt c and cleaved-caspase3 expression in the figure l. **p** Representative images of immunofluorescence double labeling of Tom20 and Bax in different kidney tissues. *P < 0.05 vs. db/m, ^#^*P* < 0.05 vs. db/db.

Next, we tested the effect of *t*-AUCB on mitochondrial function in db/db mice. First, we found that *t*-AUCB administration partially reversed the loss of ΔΨm (Fig. 7a) and significantly alleviated the production of mitochondrial ROS in the kidneys of db/db mice (Fig. 7b). In addition, we found that *t*-AUCB treatment markedly decreased the expression of Drp1 and increased the expression of Mfn2 in the kidneys of db/db mice (Fig. 7c), as reflected by Western blotting. Moreover, oil red O staining showed increased abnormal lipid accumulation in the kidney tissues of db/db mice (Fig. 7h), which was accompanied by decreased expression of fatty acid oxidation (FAO)-regulating proteins including peroxisome proliferator-activated receptor γ coactivator-1α (PGC-1α) and carnitine palmitoyltransferase 1 A (CPT1A) (Fig. 7i). However, *t*-AUCB treatment significantly attenuated lipid accumulation and upregulated the FAO-regulating protein expression of PGC-1α and CPT1A in the kidneys of db/db mice. Furthermore, we observed that *t*-AUCB administration significantly decreased the expression of Bax, Cyt c and cleaved -caspase3 (Fig. 7l) and also attenuated the colocalization of Tom20 and Bax in the kidneys of db/db mice (Fig. 7p).

### *t*-AUCB administration attenuated ER stress in the kidneys of db/db mice

**Fig. 8:**
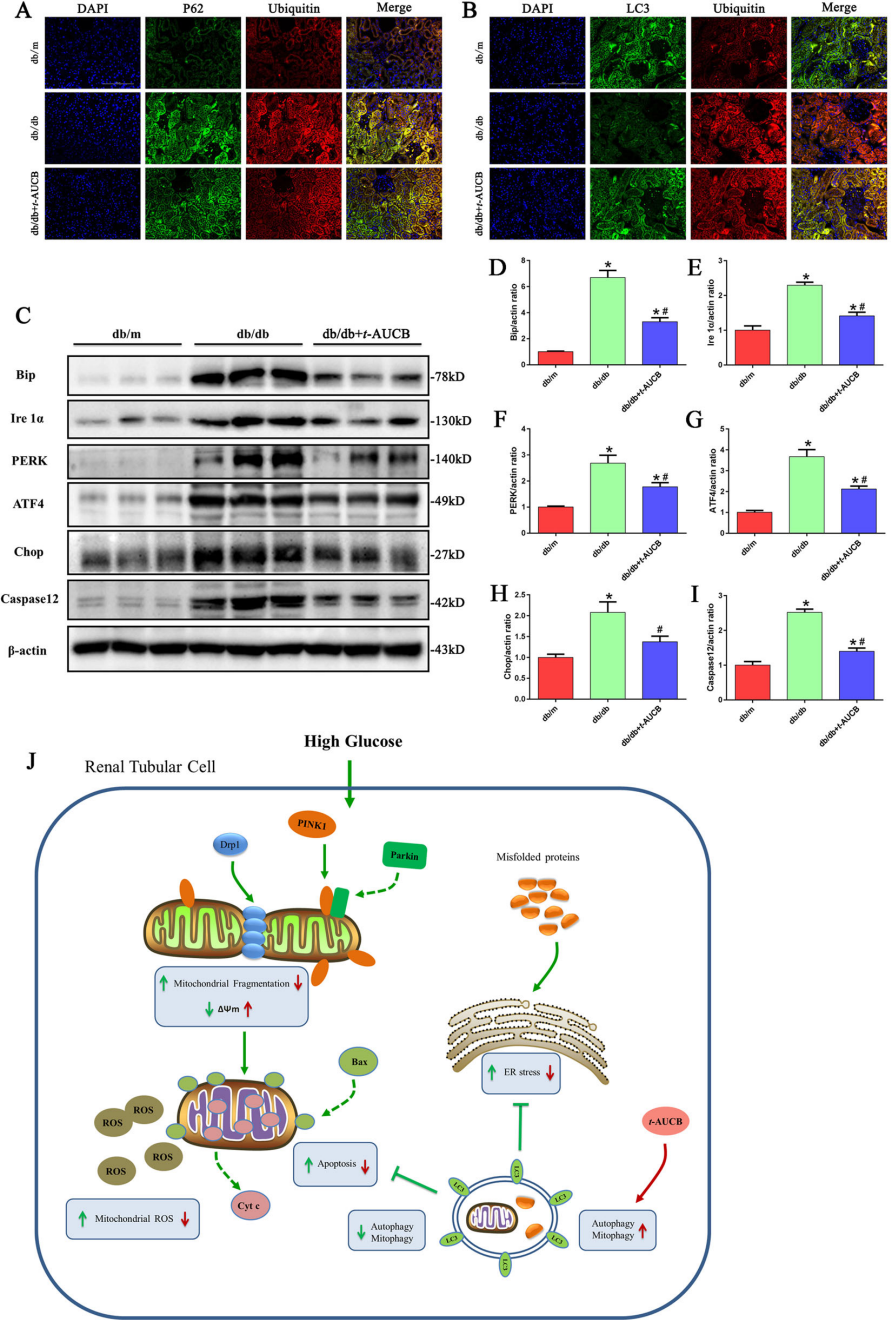
*t*-AUCB administration attenuated ER stress in db/db mice. **a**, **b** Representative images of immunofluorescence double labeling of p62 and Ubiquitin as well as LC3 and Ubiquitin in different kidney tissues. **c** Western blot analysis of Bip, Ire1α, PERK, ATF4, Chop and Caspase12 expression in kidney tissues. **d–i** Densitometric analysis of Bip, Ire1α, PERK, ATF4, Chop and Caspase12 expression in figure c. **j** Schematic diagram depicting inhibition of soluble epoxide hydrolase with *t*-AUCB attenuates renal tubular mitochondrial dysfunction and ER stress by restoring autophagic flux in diabetic nephropathy. HG exposure induced mitochondrial dysfunction, and ER stress could then lead to cell apoptosis and injury. Under normal conditions, autophagy serves as an adaptive mechanism to eliminate damaged mitochondria (mitophagy) and misfolded proteins to maintain mitochondrial quality and alleviate ER stress. However, under HG conditions, both autophagy and mitophagy are suppressed, and a compromise in the process of PINK1/Parkin -mediated mitophagy results in insufficient autophagic removal of mitochondria, which eventually leads to the accumulation of fragmented and damaged mitochondria, and subsequently triggers ROS overproduction and activation of the mitochondrial apoptotic pathway. Meanwhile, impaired autophagy hinders autophagic function as a compensatory mechanism to remove misfolded protein aggregates, which enhances the accumulation of ubiquitinated protein and aggravates ER stress. Interestingly, pharmacologic inhibition of sEH by *t*-AUCB restores impaired autophagy flux, which subsequently attenuates renal tubular mitochondrial dysfunction and ER stress, thereby alleviating hyperglycemia-induced proximal renal tubular injury.

Finally, we determined the role of *t*-AUCB on ER stress in the kidneys of db/db mice. As shown in Fig. 8a, we found that a substantial amount of ubiquitinated proteins accumulated in the renal tubules of db/db mice, which was accompanied by upregulated expression of the following ER stress-related proteins: Bip, Ire1α, PERK, ATF4, Chop and Caspase12 (Fig. 8c). However, *t*-AUCB administration significantly increased the colocalization of LC3 with ubiquitinated proteins (Fig. 8b) and attenuated the accumulation of ubiquitinated proteins and the expression of reticulum stress-related proteins in the kidneys of db/db mice (Fig. 8c). Similarly, immunofluorescence analysis showed that db/db mice treated with *t*-AUCB exhibited significant attenuation of Bip and Chop expression compared with that of the controls, as evidenced by decreased fluorescence density (Supplement Fig. 4a, b).

## Discussion

Renal tubular cell dysfunction is a significant contributor to the pathogenesis and progression of DN^3,4^. Previous studies have demonstrated that mitochondrial dysfunction plays a pivotal role in the pathogenesis of DN^29^. Mitochondria are highly dynamic organelles that constantly undergo cycles of fission and fusion, and the selective autophagic clearance of damaged mitochondria (mitophagy) purifies the mitochondrial pool^30^. Therefore, mitochondrial dynamics and mitophagy are essential for performing mitochondrial quality control and maintaining mitochondrial homeostasis, and these processes have been investigated in various diseases^31–33^.

Autophagy is usually considered to be a cellular survival mechanism under various stress conditions. However, excessive, dysregulated and defective autophagy are implicated in a variety of human diseases^34^. Accumulating evidence has implicated autophagy insufficiency in the pathogenesis of DN and proposed that upregulation of autophagy might represent a plausible therapeutic intervention in DN. Indeed, previous studies have reported that autophagy is inhibited in tubule epithelial cells during both type 1 and type 2 DN^35–37^. Consistently, in this study, we found that autophagy activity was significantly suppressed in the kidneys of db/db mice and in HK-2 cells exposed to HG, as shown by a reduction in the expression of autophagy-related proteins, Lamp2, Atg5, LC3 II/I ratio, a reduction in the number of autophagosomes and/or the accumulation of the autophagy substrate p62. However, it is interesting to note that enhanced autophagy activity is also observed in animal models of DN^38,39^ and in HK-2 cells exposed to HG^40^. Thus, it would be interesting to investigate the exact regulatory mechanism of autophagy in diabetic nephropathy in future studies.

Mitophagy is a specific process that selectively removes dysfunctional or damaged mitochondria for degradation through autophagy. Mitophagy serves as an important mechanism of mitochondrial quality control, and it is largely regulated by PINK1 and Parkin signaling pathway^41^. When mitochondria are injured, PINK1 stabilizes on the outermitochondrial membrane and then recruits Parkin to mitochondria. Subsequently, Parkin ubiquitinates several mitochondrial outer membrane proteins, including TOMM20 and VDAC, to induce and facilitate the autophagic removal of damaged mitochondria^42–44^. In the current study, the expression of PINK1 and Parkin was significantly decreased in the kidneys of db/db mice and in HK-2 cells exposed to HG. Meanwhile, the numbers of cellular autophagosomes and the amount of mitophagy were reduced in HK-2 cells exposed to HG, indicating inefficient removal of damaged mitochondria. Collectively, increased mitochondrial fragmentation and reduced autophagy or mitophagy under HG conditions were observed in tubular cells in vivo and in vitro, indicating impaired mitochondrial quality control, which may inevitably result in the accumulation of dysfunctional mitochondria and excessive ROS generation and can eventually lead to cell injury and apoptosis.

Numerous studies have demonstrated that genetic deletion or pharmacological inhibition of sEH enhances the protective biological effects of EETs by stabilizing EET levels, which significantly dampens the inflammatory response^20^, attenuates histological damage, alleviates renal tubulointerstitial^24^ and cardiac fibrosis^45^, and alleviates liver injury^46^. Therefore, alterations in sEH expression and activity will likely have potential benefits in improving the renal function of DN. In this regard, we reported increased expression of sEH protein in the kidneys of db/db mice and in HK-2 cells exposed to HG. This finding is consistent with that of Bettaieb et al., who demonstrated that sEH protein expression was increased in murine kidneys under HFD- and STZ- induced hyperglycemia^38^, which is in keeping with observations that enhanced sEH expression is found in podocytes upon lipopolysaccharide challenge^47^ or in HK-2 cells exposed to urinary proteins^48^. On the other hand, some studies showed that sEH expression was decreased in the kidneys of diabetes-associated renal injury rodent models^49,50^. Reasons for the discrepancy remain to be determined but could be a consequence of either procedural differences and different durations of challenge or different animal models.

Next, to test the role of sEH in DN, db/db mice and HK-2 cells were treated with the sEH pharmacological inhibitor *t*-AUCB, and we found that *t*-AUCB treatment partially preserved renal function of db/db mice and attenuated apoptosis of HK-2 cells exposed to HG. Although inhibition of sEH is known to have beneficial effects on cell survival, little is known about its role in regulating autophagic pathways. Several previous studies have uncovered possible relationships between sEH and autophagy. Lopez-Vicario et al. reported that inhibition of sEH with *t*-TUCB modulated autophagy and the inflammatory response in liver and obese adipose tissue^51^. Zhou et al. showed that the sEH inhibitor TPPU ameliorated ethanol-induced mouse cardiac fibrosis by restoring impaired autophagic flux^45^. In the present study, we found that inhibition of sEH with *t*-AUCB enhanced the levels of autophagy and mitophagy in the kidneys of db/db mice and in HK-2 cells exposed to HG, which is shown by the increased formation of autophagosomes and fusion of autophagosome-lysosomes, indicating that autophagy flux was restored. Furthermore, we also found that inhibition of sEH with *t*-AUCB alleviated mitochondrial dysfunction and ROS generation, both in vivo and in vitro. Meanwhile, *t*-AUCB treatment partially attenuated abnormal lipid accumulation in the kidneys of db/db mice due to its benefits in improving mitochondrial FAO. Collectively, our data precisely revealed that hyperglycemia stimulation disturbed autophagic flux by suppressing autophagy activity in the proximal tubular cells, while *t*-AUCB restored autophagy flux by increasing autophagosome formation and autophagosome-lysosome fusion. Activation of mitophagy promoted the removal of damaged mitochondria, resulting in a healthier mitochondrial pool that maintained mitochondrial homeostasis following hyperglycemia stimulation, thereby preserving mitochondrial function, reducing ROS generation and promoting cell survival.

Excess misfolded protein accumulation in the ER could lead to ER stress, which subsequently activates the UPR to restore protein homeostasis and prevent aggregation^16^. The UPR is an adaptive protective mechanism during ER stress; however, prolonged or severe ER stress can ultimately lead to cell death. Previous studies have demonstrated that ER stress plays a vital role in the pathogenesis of DN^52^. In our study, enhanced ER stress was also observed in the kidneys of db/db mice and in HK-2 cells exposed to HG. Additionally, increasing evidence has demonstrated that there is an interplay between ER stress and autophagy, consistent with the view that autophagy could be an adaptive cellular mechanism combating ER stress by removing aggregated proteins^28^, our current study showed that *t*-AUCB treatment significantly increased the colocalization of LC3 with ubiquitinated aggregates and reduced the accumulation of ubiquitinated proteins, subsequently attenuating ER stress in the kidneys of db/db mice and in HG-induced HK-2 cells. Our findings are in line with a previous study, which showed that sEH deficiency or inhibition attenuated high fat diet-induced ER stress in mouse liver and adipose tissues^46^; further, these data are consistent with those reported by Harris et al., who showed that inhibition of sEH attenuated hepatic endoplasmic reticulum stress and fibrosis induced by carbon tetrachloride (CCl4) in mice^53^. Therefore, our results demonstrated that the exact mechanism of *t*-AUCB-mediated protective autophagy was involved in modulating mitochondrial function and ER stress in DN.

However, it is worth noting that not all the studies showed the beneficial effect of sEH inhibition on kidney diseases. Jung et al. reported that sEH-inhibition with *t*-AUCB failed to elicit protective effects in the 5/6 nephrectomy mouse model and notably aggravated proteinuria^54^. Thus, the role of sEH in diverse kidney diseases needs to be further elucidated by future studies.

In summary, we provided evidence revealed that hyperglycemia -induced mitochondrial dysfunction, ER stress and impaired autophagic flux in the renal proximal tubular cells. Inhibition of sEH with *t*-AUCB attenuated hyperglycemia -mediated renal injury by restoring autophagic flux, which consequently resulted in improved mitochondrial function and reduced ER stress (Fig. 8j). Our observations highlight the potential of sEH pharmacological inhibition as a therapeutic approach in DN.

## Materials and methods

### Cell culture and treatment

HK-2 cells (human proximal tubular epithelial cells) was a kind gift from Professor Ruan (The Centre for Nephrology, Royal Free and University College Medical School, London, United Kingdom).The cells were cultivated in DMEM/F-12 medium supplemented with 10% fetal bovine serum (FBS, Gibco), 1000 U/L penicillin and 100 μg/mL streptomycin at 37°C in 5% CO_2_ air. The cells were treated with low glucose (LG, 5.5 mM d-glucose), high-glucose (HG, 30 mM d-glucose), or osmotic control conditions (5.5 mM glucose+24.5 mM D-mannitol) for 24 h. To pharmacologically inhibit sEH, HK-2 cells were treated with LG or HG for 24 h with or without 10 μM sEH inhibitor trans-4-(4-(3-adamantan-1-yl-ureido)-cyclohexyloxy)-benzoic acid (*t*-AUCB, ApexBio,USA).

### Animals

Four-week-old male db/db mice and db/m mice were purchased from the Mode Animal Research Center of Nanjing University (Nanjing, China). The animals were housed in the Animal Center of Chongqing Medical University and kept under a 12-hour light/12-hour dark cycle with free access to water and food. After 1 week of acclimatization, they were randomly divided by random number table into the following three groups for the animal experiments: a db/m group (control, *n* = 10), a db/db group (*n* = 10) and a db/db+sEH inhibitor group (db/db mice treated with *t*-AUCB, *n* = 10). At 16 weeks, the mice in the db/db + *t*-AUCB group were treated daily with *t*-AUCB at a dose of 2 mg/L (the compound *t*-AUCB was first solubilized in dimethyl sulfoxide (DMSO) and was subsequently immediately added to drinking water) in drinking water for 4 weeks before being sacrificed. All procedures were conducted in accordance with the relevant institutional guidelines of the Ethics Committee of Chongqing Medical University.

### Measurement of lipid accumulation

Oil red O staining was used to measure lipid accumulation. Frozen kidney sections were thawed at room temperature for 10 min and then were rinsed in PBS. Then, kidney tissues were stained with an Oil red O working solution for 30 min at room temperature before being pretreated with 60% isopropanol for 5 s; then they were treated with hematoxylin for 5 min. After three washes with PBS, images were visualized under a fluorescence microscope.

### Detection of intracellular and mitochondrial ROS production

To assess intracellular and mitochondrial ROS production, the treated cells were incubated with DCFH-DA (10 μM) and MitoTracker red (50 nM) in serum-free culture medium at 37 °C for 30 min, or the cells were incubated with 5 μM MitoSOX Red (M36008, Invitrogen, USA) and 50 nM MitoTracker green (C1048, Beyotime, China) in serum-free culture medium at 37 °C for 20 min. Mitochondrial ROS production in kidney tissues was assessed using 4-μm-thick frozen sections and staining for MitoSOX. After three washes with PBS, the images were observed under a fluorescence microscope.

### Assessment of mitochondrial transmembrane potential (∆Ψm)

The mitochondrial membrane potential of HK-2 cells and kidney tissue was measured using JC-1 fluorescence dye (Beyotime, China) according to the manufacturer’s instructions. Briefly, the treated cells or frozen kidney sections were incubated with JC-1 at 10 μg/ml for 20 min in the dark at 37 °C, and then they were washed with PBS. The images were immediately observed by fluorescence microscopy. Increased green fluorescence levels and decreased red fluorescence levels indicated a potential collapse of the mitochondrial membrane.

### GFP-LC3 lentivirus transfection

A GFP-LC3 lentivirus was purchased from Genechem (LV-MAP1LC3B, 3905-1). For transfection experiments, HK-2 cells were seeded into 12-well plates and infected with a GFP-LC3 lentivirus at an optimal MOI of 20 according to the manufacturer’s instructions. After 48 h of infection, the cells were screened with puromycin, and the resistant cells were used to perform subsequent experiments.

### Monodansylcadaverine (MDC) staining

MDC is an autofluorescent agent that has been utilized to label autophagic vacuoles. Briefly, HK-2 cells were seeded on glass coverslips, and then the treated cells were incubated with 50 μM MDC (Sigma-Aldrich, USA) in serum-free medium at 37 °C for 15 min in the dark. After staining, the cells were washed three times with PBS and were immediately observed with a fluorescence microscopy.

### Western blot analysis

For Western blot analyses, total protein was extracted by lysis buffer (Beyotime, China), and the protein from kidney tissues and HK-2 cells was separated by 8%–12% SDS-PAGE. The fractionated proteins were transferred onto PVDF membranes (Millipore), which were then blocked with 5% nonfat milk for 3 h. The PVDF membranes were then incubated with the following primary antibodies: mouse anti-sEH (1:1000, Santa Cruz, sc-166961), rabbit anti-Bip (1:1000, Abcam, #21685), rabbit anti-Ire1α (1:1000, CST, #3294), rabbit anti-PERK (1:1000, CST, #3192), rabbit anti-ATF4 (1:1000, CST, #11815), rabbit anti-Caspase12 (1:1000, Abcam, #62484), mouse anti-Chop (1:1000, CST, #2895), rabbit anti-cleaved -caspase3 (1:1000, CST, #9664), rabbit anti-Cytochrome c (1:1000, Santa Cruz, sc-13156), rabbit anti-Bax (1:1000, CST, #2772), rabbit anti-Lamp2 (1:1000, Abcam, #13524), rabbit anti-Atg5 (1:1000, Abcam, # 108327), rabbit anti-LC3 (1:1000, CST, #4108), mouse anti-p62 (1:1000, abcam, #109012), rabbit anti-Parkin (1:1000, Abcam, #77924), mouse anti-PINK1(1:1000, abcam, #186303), rabbit anti-PGC-1α (1:1000, Abcam, #54481), rabbit anti-CPT1A (1:1000, Abcam, #128568) and mouse anti-β-actin (1:5000, Sungene Biotech, # KM9001T). After overnight incubation, the membranes were incubated with secondary antibodies (MultiSciences Biotech) at room temperature for 1 h. Membrane blot signals were then visualized with an ECL chemiluminescence system (GE Healthcare, Piscataway, NJ, USA). The density of labeled protein bands was quantified by Quantity One software, and the results were normalized to β-actin.

### Immunofluorescent staining

HK-2 cells were seeded on glass coverslips, and then the treated cells were fixed with 4% paraformaldehyde for 30 min, permeabilized with 0.1% Triton X-100 for 3 min and blocked with 5% BSA for 1 h at room temperature. Then, the cells were incubated with the following primary antibodies: mouse anti-p62 (1:200), rabbit anti-LC3 (1:200), rabbit anti-Bax (1:200) or mouse anti-Lamp2 (1:200) at 4 °C overnight. After washing with PBS, they were incubated with Alexa Fluor 488 goat anti-rabbit IgG (1:400, Invitrogen, USA) secondary antibodies for 1.5 h at 37 °C. Cells were stained with DAPI to delineate the nuclei, and then they were examined by fluorescence microscopy.

For kidney tissue, paraffin-embedded kidney tissue sections were deparaffinized in xylene and rehydrated with a graded alcohol series. Antigen retrieval was performed by microwave treatment in 10 mmol/L sodium citrate buffer (pH 6.0) for 15 min, and endogenous peroxidase activity was inactivated by treatment with 0.3% H2O2 for 15 min. After washing, the sections were blocked with normal goat serum and then were incubated at 4 °C overnight with the following primary antibodies: mouse anti-p62 (1:200), rabbit anti-LC3 (1:200), rabbit anti-Bax (1:200), mouse-Lamp2 (1:200), mouse anti-Chop (1:200), rabbit anti-Bip (1:200), mouse anti-Tom20 (1:200), mouse anti-VDAC (1:200), mouse anti-PINK1 (1:200) or mouse anti-sEH (1:200). After washing with PBS three times, sections were incubated for 1.5 h at 37 °C with secondary antibody Alexa Fluor 488 goat anti-rabbit IgG (1:400, Invitrogen, A11008, USA) or Alexa Fluor 488 goat anti-mouse IgG (1:400, Invitrogen,A11001, USA). Then the slides were stained with DAPI for 3 min and were visualized by fluorescence microscopy.

### Measurement of sEH enzymatic activity

EETs can be hydrolyzed to DHETs by the sEH enzyme, so the ratios of 14,15-EET to 14,15-DHET can indirectly represent the enzymatic activity of sEH. The level of 14,15-EET and 14,15-DHET, 14,15-EET to 14,15-DHET ratios of kidney and HK-2 cells were assessed by a 14,15-EET/14,15-DHET ELISA kit (Jianglai Biological, Shanghai), according to the manufacturer’s instructions.

### Analysis of cell apoptosis

Apoptosis was analyzed by an Annexin V-FITC/PI apoptosis assay kit (Sungene Biotech, China) as previously described^55^.

### Statistical analysis

Data are reported as the means ± SEM at least three independent experiments. The significance of the differences between 2 groups was analyzed using Student’s *t*-tests, and multiple comparisons were performed by one-way analysis of variance (ANOVA) followed by the Tukey post hoc test. For animal studies, sample size was estimated according to previous studies, which perform similar experiments to detect significant difference between samples. The variance is similar between groups that are being statistically compared. GraphPad Prism software was used for all statistical analyses. Differences were considered statistically significant at *P* < 0.05.

## Supplementary information

Supplementary figure legends

Supplement Fig 1

Supplement Fig 2

Supplement Fig 3

Supplement Fig 4

Author Contribution

Reproducibility Checklist
