# Supplementary material for: Inhibition of soluble epoxide hydrolase attenuates renal tubular mitochondrial dysfunction and ER stress by restoring autophagic flux in diabetic nephropathy
Source: Cell Death Dis. 2020 May 21;11(5):385. doi: 10.1038/s41419-020-2594-x (PMC7242354; doi:10.1038/s41419-020-2594-x)
Supplement: Supplementary file 1 — Supplementary figure legends [file 41419_2020_2594_MOESM1_ESM.docx]

**Supplementary figure legends**

Supplement Figure 1. *t*-AUCB inhibited sEH enzymatic activity in HK-2 cells exposed to HG. (A) The levels of 14,15-EET in different groups supernatant of HK-2 cells after treated with HG or/and *t*-AUCB for 24 h. (B) The levels of 14,15-DHET in different groups supernatant of HK-2 cells after treated with HG or/and *t*-AUCB for 24 h.(n=3,^*^*P*< 0.05 vs. LG, ^#^*P*< 0.05 vs. *t*-AUCB, ^Δ^*P*< 0.05 vs. HG.)

Supplement Figure 2. Inhibition of sEH attenuated HG induced Bax and Cyt c redistribution in HK-2 cells. (A-B) Representative images of immunofluorescence double labeling of Bax and MitoTracker Red or Cyt c and MitoTracker Red in different groups of HK-2 cells after treated with HG or/and *t*-AUCB for 24 h.

Supplement Figure 3. *t*-AUCB inhibited sEH enzymatic activity in the kidney of db/db mice. (A) The levels of 14,15-EET in the urine of db/m, db/db and db/db mice treated with *t*-AUCB. (B) The levels of 14,15-DHET in the urine of db/m, db/db and db/db mice treated with *t*-AUCB.^*^*P*< 0.05 vs. db/m, ^#^*P*< 0.05 vs. db/db.

Supplement Figure 4. *t*-AUCB administration attenuated ER stress in db/db mice. (A-B) Representative images of immunofluorescence staining of Bip and Chop in kidney tissues. (C-D) Quantification of the fluorescence intensity of Bip and Chop in Figure A and B. ^*^P< 0.05 vs. db/m, ^#^P< 0.05 vs. db/db.
