# Supplementary figures and images for: Inhibition of soluble epoxide hydrolase attenuates renal tubular mitochondrial dysfunction and ER stress by restoring autophagic flux in diabetic nephropathy
Source: Cell Death Dis. 2020 May 21;11(5):385. doi: 10.1038/s41419-020-2594-x (PMC7242354; doi:10.1038/s41419-020-2594-x)

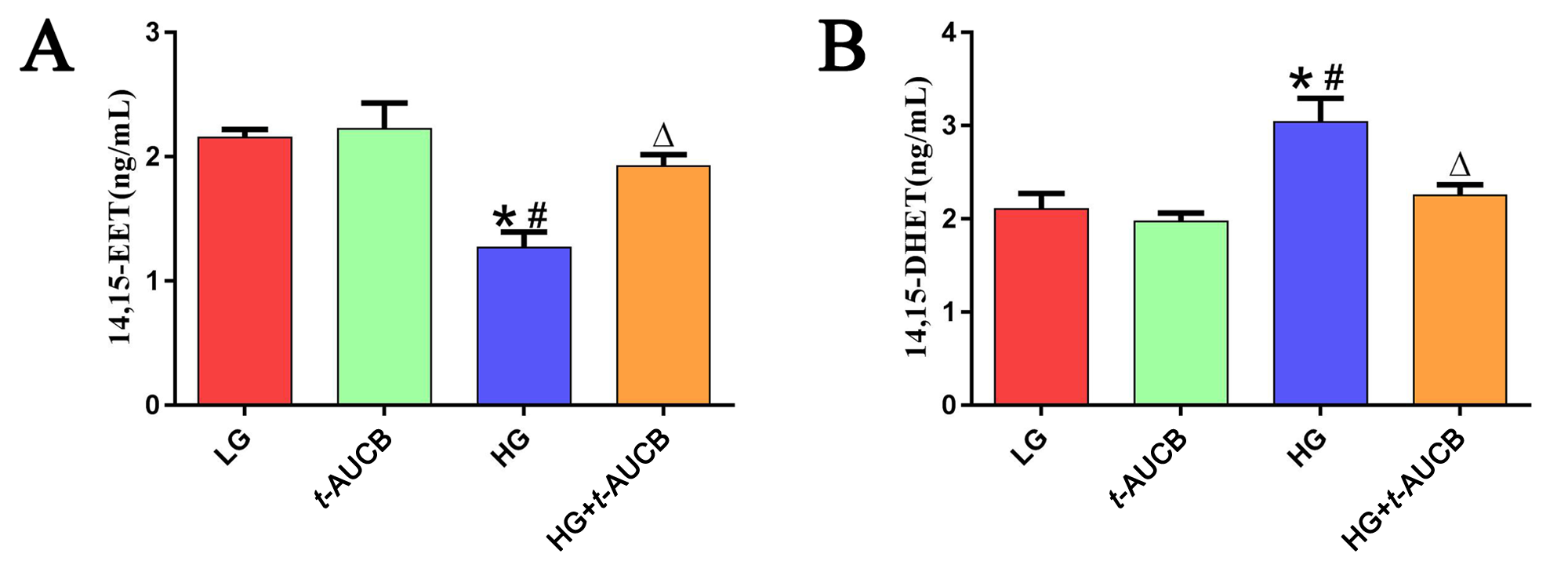

Supplement: Supplementary file 2 — Supplement Fig 1 [file 41419_2020_2594_MOESM2_ESM.png]

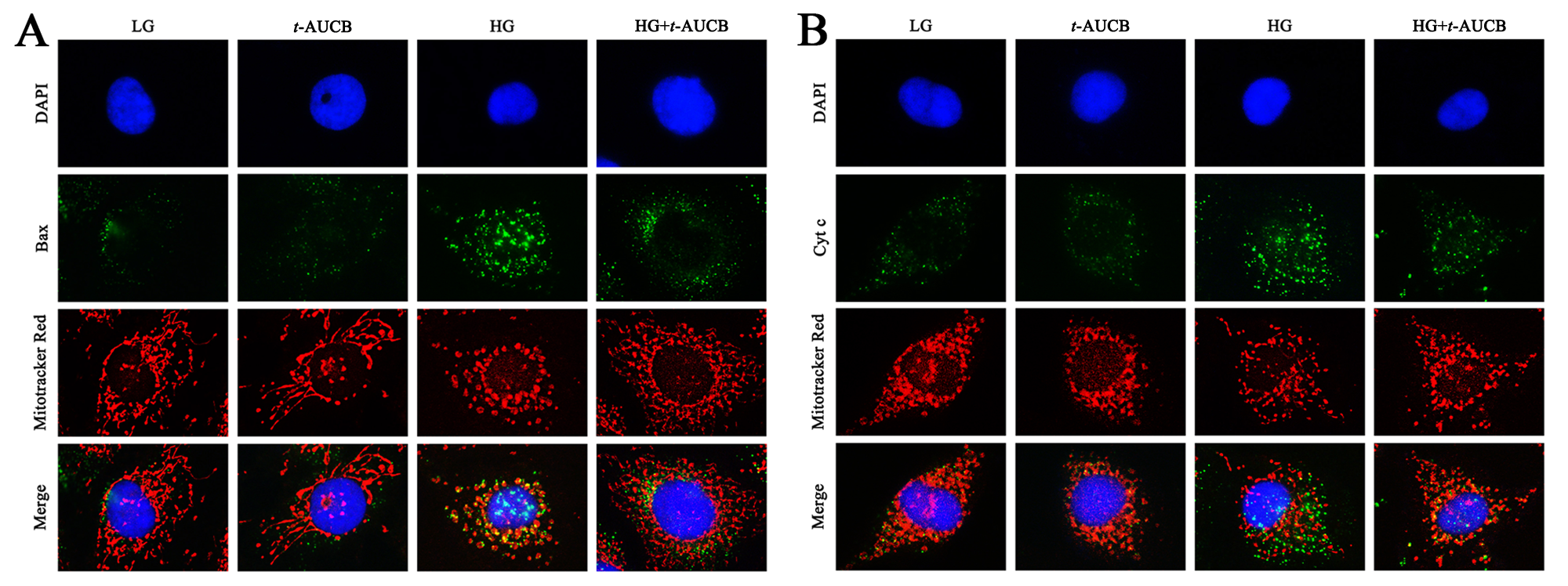

Supplement: Supplementary file 3 — Supplement Fig 2 [file 41419_2020_2594_MOESM3_ESM.png]

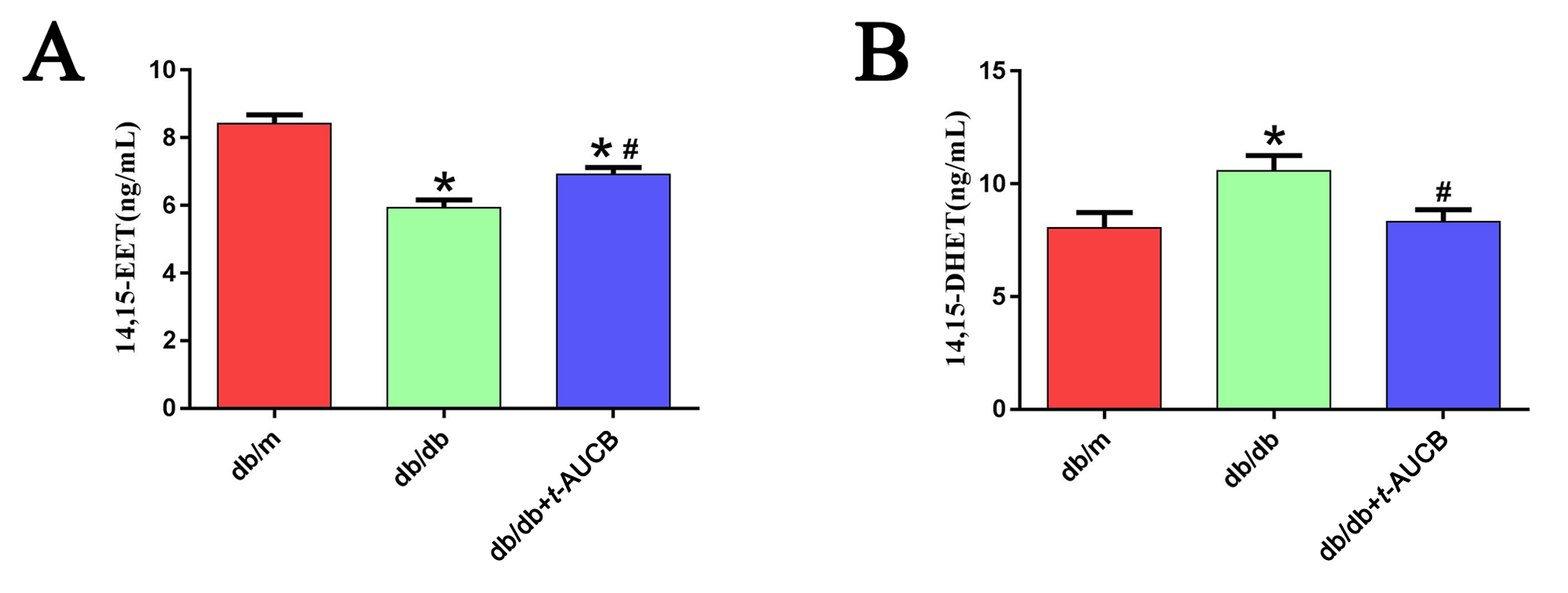

Supplement: Supplementary file 4 — Supplement Fig 3 [file 41419_2020_2594_MOESM4_ESM.png]

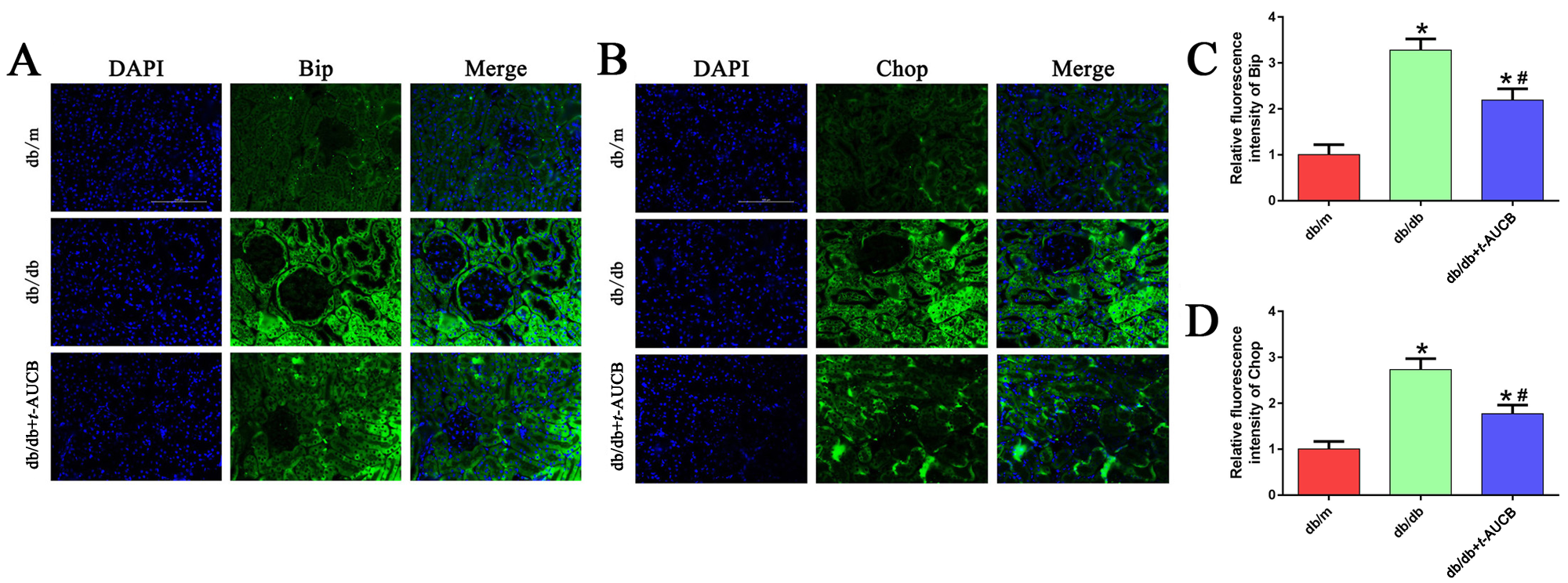

Supplement: Supplementary file 5 — Supplement Fig 4 [file 41419_2020_2594_MOESM5_ESM.png]
