# Supplementary material for: Inhibition of soluble epoxide hydrolase attenuates renal tubular mitochondrial dysfunction and ER stress by restoring autophagic flux in diabetic nephropathy
Source: Cell Death Dis. 2020 May 21;11(5):385. doi: 10.1038/s41419-020-2594-x (PMC7242354; doi:10.1038/s41419-020-2594-x)
Supplement: Supplementary file 6 — Author Contribution [file 41419_2020_2594_MOESM6_ESM.pdf]

DECLARATION OF CONTRIBUTIONS TO ARTICLE

ADMC

Manuscript Number:	Journal Name:	
CDDIS-20-0267	*Cell Death & Disease*	(the 'Journal')

Proposed Title of the Contribution:

Inhibition of soluble epoxide hydrolase attenuates renal tubular mitochondrial dysfunction and ER stress by restoring autophagic flux in diabetic nephropathy	(the 'Contribution)

Author(s):

Xu-shun Jiang, Xing-yang Xiang, Xue-mei Chen, Jun-ling He, Ting Liu, Hua Gan, Xiao-gang Du	(the 'Authors')

For all *CDDis* articles, each person named as an author in the published version must be able to show he or she has contributed substantially to the article.

Authorship credit should be based on 1) substantial contributions to conception and design, acquisition of data, or analysis and interpretation of data; 2) drafting the article or revising it critically for important intellectual content; and 3) final approval of the version to be published. Authors should meet conditions 1, 2 and 3.

Any person who cannot be shown to have made a substantial contribution to the article cannot be listed as an author in the final version. The name of any person who is deemed to have made a minor contribution can, however, appear in the Acknowledgments section of the article.

Please complete the table below to indicate the contributions of all named authors to the manuscript.

Author Full Name:	Specification of Contribution to the Manuscript:
Xu-shun Jiang	Performed the experiments, analysis of data and wrote the article
Xing-yang Xiang	Performed the experiments and revising the article
Xue-mei Chen	Interpretation of data and revising the article
Jun-ling He	analysis and interpretation of data, wrote the article
Ting Liu	analysis and interpretation of data
Hua Gan	Interpretation of data and revising the article
Xiao-gang Du	Conceived and designed the experiments

Please complete the table below to indicate the contributions of all named authors to the figures.

Figure 1:

Xu-shun Jiang : Performed the experiments
Xing-yang Xiang: Performed the experiments
Ting Liu: analysis and interpretation of data
Xiao-gang Du: Designed the experiments

Figure 2:

Xu-shun Jiang : Performed the experiments
Xing-yang Xiang: Performed the experiments
Xue-mei Chen: Interpretation of data
Jun-ling He: analysis and interpretation of data
Xiao-gang Du: Designed the experiments

Figure 3:

Xu-shun Jiang : Performed the experiments
Xing-yang Xiang: Performed the experiments
Xue-mei Chen: Interpretation of data
Jun-ling He: analysis of data
Xiao-gang Du: Designed the experiments

Figure 4:

Xu-shun Jiang : Performed the experiments
Xing-yang Xiang: Performed the experiments
Xue-mei Chen: Interpretation of data
Jun-ling He: analysis and interpretation of data
Xiao-gang Du: Designed the experiments

Figure 5:

Xu-shun Jiang : Performed the experiments
Xing-yang Xiang: Performed the experiments
Xue-mei Chen: Interpretation of data
Ting Liu: analysis of data
Xiao-gang Du: Designed the experiments

Figure 6:

Xu-shun Jiang : Performed the experiments
Xing-yang Xiang: Performed the experiments
Hua Gan: Interpretation of data
Jun-ling He: analysis and interpretation of data
Xiao-gang Du: Designed the experiments

Signed for and on behalf of the Author(s):	Print Name:	Date:
Xiao-gang Du	Xiao-gang Du	2020-01-26
